# Tuber starch amylose content is associated with cold-induced sweetening in potato

**DOI:** 10.1002/fsn3.137

**Published:** 2014-06-30

**Authors:** Shelley H Jansky, Diego A Fajardo

**Affiliations:** USDA-ARS, Vegetable Crops Research Unit and Department of Horticulture, University of Wisconsin-MadisonMadison, Wisconsin

**Keywords:** Processing quality, *Solanum tuberosum*, starch granule

## Abstract

Cold-induced sweetening (CIS) is the accumulation of reducing sugars in potato tubers at low storage temperatures. It is undesirable because it results in dark fry products. Our study evaluated the relationship between genetic resistance to CIS and two starch parameters, amylose content and starch granule size. We found that the amylose content in four CIS-resistant varieties was higher than that in five susceptible varieties. Amylose content was influenced not only by variety but also storage, production year, and field location. However, interactions between amylose content and environmental variables were not detected. In contrast, starch granule size was not associated with CIS resistance. No effect of storage on starch granule size was detected, and interactions among variety, production year, and field location were observed. Tuber starch amylose content should be considered a source of variability for CIS.

## Introduction

Worldwide, most potatoes are produced in temperate regions, where the bulk of the crop is harvested during a narrow window of time in the fall. Even in tropical regions where year round agriculture is possible, potato is often grown in one season, in rotation with other crops during the year. Consequently, most of the crop is harvested in a short period of time and must be stored to provide a consistent supply throughout the year. Tubers are typically stored at cold temperatures to reduce shrinkage due to respiration and to minimize losses to tuber-borne pathogens. Cold-stored tubers, however, accumulate the reducing sugars glucose and fructose (Fitzpatrick and Porter 1966; Schippers 1975; Ewing et al. 1981). When these tubers are fried at high temperatures to produce potato chips or French fries, the sugars interact with amino acids in the Maillard reaction to produce an unacceptably dark colored product. The accumulation of reducing sugars during cold storage, called cold-induced sweetening (CIS) is a heritable trait (Hayes and Thill 2002, 2003; Menendez et al. 2002; Jansky and Hamernik 2009; Jansky et al. 2011). A few key starch metabolism enzymes have been found to be associated with CIS (Li et al. 2005, 2008; Bhaskar et al. 2010; Wu et al. 2011). Until the development of CIS-resistant cultivars, the potato-processing industry sometimes reconditioned tubers to decrease the amount of reducing sugars and improve the quality of fried products after cold storage of tubers (Fitzpatrick and Porter 1966; Schippers 1975). Reconditioning is achieved by warming the tubers for one to several weeks at 12–15°C.

Studies of the effects of cold temperature storage on processing quality have focused on the activity of enzymes involved in the conversion of starch to sugars. However, Ohad et al. (1971) suggested that cold storage temperatures may also damage the amyloplast membrane. This would make the membrane more permeable to starch hydrolysis enzymes. Starch properties, such as the amylose:amylopectin ratio, may also influence starch hydrolysis rates in granules, where starch is stored in tubers. This, in turn, would have an impact on the conversion of starch to free sugars (Barichello et al. 1990). In a previous study, the amylose content in the starch of the CIS-resistant variety ND860-2 was found to be higher than that of the susceptible line Norchip (Barichello et al. 1990). In addition, the diameters of starch granules of ND860-2 were more stable during cold storage than those of Norchip. Based on this comparison of one pair of varieties, the authors concluded that starch granule composition contributes to the CIS phenotype. That study was followed by another one that evaluated two resistant and two susceptible cultivars and found no association between CIS resistance and amylose content (Cottrell et al. 1995). We were interested in following up with a broader array of varieties to evaluate the association between starch granule properties and CIS resistance.

For this study, we chose four genetically diverse varieties with documented high levels of resistance to CIS, M3, and M5 (Jansky et al. 2011), ND860-2 (Coffin et al. 1987), and White Pearl (Groza et al. 2006). They were compared to five cultivars that are susceptible to CIS, Atlantic, Jacqueline Lee, Katahdin, Superior, and Yukon Gold. Atlantic and Superior are used commercially for chip production. We grew the varieties across 3 years and two locations, and evaluated amylose content in fresh, stored, and reconditioned tubers and starch granule size in fresh and stored tubers.

## Materials and Methods

The nine tetraploid varieties (M3, M5, ND860-2, Atlantic, Jacqueline Lee, Katahdin, Superior, White Pearl, and Yukon Gold) were grown at the Hancock, Wisconsin, Agricultural Experiment Station in 2010, 2011, and 2012. They were also grown in a field plot near Antigo, Wisconsin, in 2011 and 2012. The trial was not carried out in Antigo in 2010 because there was insufficient seed available. The Hancock trial was planted on 4 May 2010, 8 June 2011, and 8 May 2012, while the Antigo trial was planted on 23 May 2011 and 10 May 2012. Vines were chemically killed in late August and plots were harvested at Hancock on 3 September 2010, 6 October 2011, and 11 September 2012, and at Antigo on 3 October 2011 and 2 October 2012. In all trials, two replications of five plant plots were grown for each variety in a randomized complete block design. At harvest, 10 medium size tubers were randomly selected. Two were processed (peeled, diced, placed in a −80°C freezer, and lyophilized) within 1 week of harvest. These comprised the fresh sample. Four tubers were stored at 4°C and four were stored at 6°C. After 50 days, the tubers were removed from cold storage. Two tubers from each storage temperature were processed as described above to provide tissue for the stored treatment. The remaining two tubers from each storage temperature were reconditioned at 18°C for 34 days and then processed to provide tissue for the reconditioning treatment. Lyophilized tuber samples were ground in a Wiley mill with a 40 mesh screen.

The relative proportions of amylose and amylopectin in lyophilized starch samples were obtained by applying a method based on the protocol published by Hovenkamp-Hermelink et al. (1988) with some modifications (Fajardo et al. 2013b). The amount of starting material was scaled down and ground freeze-dried tubers were used. A 20–30 mg sample of tuber tissue was diluted and mixed in 500 *μ*L 45% (w/v) perchloric acid. After a 4-min incubation period at room temperature, 16 mL of distilled water was added to the solution and mixed by vortexing. After nonsoluble material settled to the bottom of the tube, 40 *μ*L of solution was transferred to a microtiter plate (avoiding the pipetting of any particles) and mixed with 50 *μ*L of Lugol's iodine solution (6 g KI + 0.4 g I_2_ in 2 mL of water). Each sample was mixed by pipetting and the plate was placed on a Bio Tek ELX800 microplate spectrophotometer (Winooski, VT). Absorbances at 550 and 620 nm were read immediately. Percent amylose was determined after comparing the amylose to amylopectin ratio of each sample (620 nm absorbance/550 nm absorbance) with a standard curve generated from amylose (Sigma No. 859656, St. Louis, MO) and amylopectin (Sigma No. A8515) solutions at different concentrations (see Hovenkamp-Hermelink et al. 1988). Samples from two different tubers collected from each of the two field replications were evaluated, and each sample was extracted twice for amylose determination.

The freeze-dried tuber samples used for amylose determination were also used to measure starch granule surface area of the two varieties with the highest amylose content (M5 and ND860-2) and the lowest amylose content (Atlantic and Yukon Gold). Samples from fresh and 6°C stored tubers collected at Hancock and Antigo in 2011 and 2012 were evaluated. Each sample was added to a microscope slide and stained with iodine. Four fields of view of each sample were photographed at 100× magnification. Then, using digital imaging software (ImageJ; Rasband 1997–2014), the surface area of each starch granule in three fields of view from each of two slides was determined. In total, 38,197 starch granules were measured.

Percent amylose and mean starch granule surface area were analyzed using the general linear model procedure in SAS (version 9.3; SAS Institute, Cary, NC) with a model including effects for variety, treatment (fresh, stored, reconditioned), field location, year, and interactions among the main effects. Means separation was carried out using Fisher's protected least significant difference (LSD) test at*P* = 0.05.

## Results and Discussion

Interactions among the main effects (treatment, location, year, and variety) for percent amylose in tuber starch were not significant, so analysis of variance was carried out on the entire data set. There was a significant effect of variety (*P* < 0.0001) on amylose content, with the highest amylose levels in the CIS-resistant varieties (Table1). The CIS-susceptible cultivar Jacqueline Lee was not significantly different than the CIS-resistant varieties, but otherwise the amylose content in each resistant variety was higher than that of each susceptible variety.

**Table 1:** Effect of variety on percent amylose in potato tuber starch.

Variety	CIS	Percent amylose
M5	Resistant	30.97a
ND860-2	Resistant	30.66a
White Pearl	Resistant	30.61a
M3	Resistant	30.35a
Jacqueline Lee	Susceptible	30.26ab
Katahdin	Susceptible	29.58bc
Superior	Susceptible	29.17c
Atlantic	Susceptible	28.92c
Yukon Gold	Susceptible	28.89c

Numbers followed by the same letter are not significantly different based on an LSD test, with*P* = 0.05. The CIS column indicates whether the variety is resistant or susceptible to cold-induced sweetening.

It is interesting and significant that the CIS-resistant varieties had higher percent amylose than the susceptible varieties (Table1). This supports the previous report by Barichello et al. (1990), in which the CIS-resistant variety ND860-2 had higher amylose levels than the susceptible cultivar Norchip. In that study, only one resistant variety was compared with one susceptible variety. Consequently, broad claims regarding the association between CIS resistance and amylose content were tenuous. Our study provides support for a physiological basis of the relationship between amylose content and CIS resistance. High amylose starch is less susceptible to hydrolysis by*α*-amylase (Barichello et al. 1990) and more stable due to hydrogen bonds within and between molecules (Biliaderis et al. 1980, 1981). In contrast to our study and that of Barichello et al. (1990), another study comparing two CIS-resistant and two CIS-susceptible cultivars found no relationship between CIS resistance and amylose content (Cottrell et al. 1995). It is possible that other factors, such as the activity of starch degradation enzymes, outweighed the effect of amylose content on CIS. Previous studies have also revealed an effect of cultivar on amylose content, but distinctions between CIS-resistant and CIS-susceptible varieties were not made (Fajardo et al. 2013a; Šimková et al. 2013).

There was a significant effect of storage treatment (*P* < 0.0001) on percent amylose, with higher amylose contents in tubers stored at 6°C, with or without reconditioning, than the other three treatments (fresh tubers, stored at 4°C, stored at 4°C and then reconditioned) (Table2). Within each treatment, when varieties were grouped by CIS resistance, the amylose content in the starch of the resistant varieties was always higher than that of the susceptible varieties (Table3). The difference was significant at*P* = 0.05 in all comparisons except the samples stored at 4°C. That comparison had a*P* value of 0.058, near the threshold for significance. The increase in the proportion of amylose in tuber starch after storage at 6°C is consistent with previous work comparing fresh and cold-stored tubers (Johnston et al. 1968; Weaver et al. 1978). An increase in the proportion of amylose during storage could occur if amylopectin was hydrolyzed at higher rate than amylose. This seems likely, since amylopectin degrades more easily than amylose and is degraded by more enzymes (Bach et al. 2013). Another explanation is that the starch synthesized during storage is higher in amylose than that synthesized during tuber development. In contrast to our study and those listed above, Golachowski (1985) and Schwimmer et al. (1954) reported that amylose content did not change after storage for 3 months at 4°C. Golachowski (1985) reported a decrease in amylose content in tubers stored at 0, 8, or 20°C. While previous studies have not evaluated amylose content in reconditioned tubers, our study found that amylose content in reconditioned tubers was the same as that of stored tubers.

**Table 2:** Effect of storage treatment on percent amylose in potato tuber starch.

Treatment	Percent amylose
Stored 6°C, reconditioned	30.62a
Stored 6°C	30.55a
Stored 4°C, reconditioned	29.73b
Stored 4°C	29.50b
Fresh	29.27b

Numbers followed by the same letter are not significantly different based on an LSD test, with*P* = 0.05.

**Table 3:** Comparison of CIS-resistant and CIS-susceptible varieties for percent amylose in potato tuber starch.

Treatment	CIS resistant	CIS susceptible
Stored 6°C, reconditioned	31.56a	29.90b
Stored 6°C	31.26a	29.98b
Stored 4°C, reconditioned	30.85a	28.84b
Stored 4°C	29.89a	29.19a
Fresh	29.73a	28.91b

Within a row, numbers followed by the same letter are not significantly different based on an LSD test, with*P* = 0.05. CIS, cold-induced sweetening.

There was a significant effect of location (*P* < 0.0001) on amylose content, with a higher mean at Hancock than Antigo (Table4). This analysis considered only 2011 and 2012, since data were not collected in Antigo in 2010. At Hancock, there was also a significant effect of year (*P* < 0.0001) on amylose content, with higher means in 2012 and 2010 than in 2011 (Table4). At Antigo, amylose content was higher in 2012 than in 2011. The significant effects of year and location indicate that environment influences amylose content to some extent. Previous studies also detected differences due to production year, which were statistically significant in some cases (Vokal et al. 2007) and not in others (Bach et al. 2013; Fajardo et al. 2013a; Šimková et al. 2013). Similarly, the effect of location has been reported in some studies (Cottrell et al. 1995; Šimková et al. 2013) but absent in others (Bach et al. 2013; Fajardo et al. 2013a). Differences among studies are likely due, in part, to the magnitude of environmental variation among sites within a study. Šimková et al. (2013) reported that three high-altitude sites produced tubers with similar amylose levels, while tubers from lower altitude sites had lower amylose content.

**Table 4:** Effect of field location and production year on potato tuber starch amylose content.

Environment	Percent amylose
Location	
Hancock	30.13a
Antigo	29.18b
Year (Hancock)	
2012	32.12a
2010	31.99a
2011	28.12b
Year (Antigo)	
2012	30.03a
2011	28.27b

The location comparison was based on data collected at Hancock and Antigo in 2011 and 2012, since data were not collected at Antigo in 2010. Numbers followed by the same letter are not significantly different based on an LSD test, with*P* = 0.05.

The lack of genotype by environment interactions for amylose content is desirable. These types of interactions are typically unpredictable and impede genetic gain from breeding. Similarly, significant effects of genotype and environment (location and year), but generally not interactions were reported for starch digestibility properties in a study that evaluated 12 potato varieties in six environments across 2 years (Bach et al. 2013).

For the starch granule size data set, analysis of variance revealed significant treatment by year (*P* = 0.0357) and location by year (*P* < 0.0001) interactions for mean starch granule surface area. Other interactions were not significant. Therefore, in subsequent analyses, starch granule size analyses were carried out separately for each year.

In 2011, there was a significant effect of variety (*P* = 0.0086) and location (*P* = 0.0176) on starch granule size. In 2012, there was also a significant effect of variety (*P* < 0.0001) and location (*P* < 0.0001). In both years, though, there was no significant difference between fresh and stored tubers for mean starch granule surface area. In contrast to amylose content, there was no difference in starch granule size between CIS-resistant and -susceptible varieties (Table5). When data from all CIS-resistant varieties were combined and compared to the combined data set from the CIS-susceptible varieties, there was no difference in starch granule surface area in 2011 (*P* = 0.3012) or 2012 (*P* = 0.7218). Starch granules from tubers grown at Hancock were smaller than those at Antigo in 2011 but larger than those at Antigo in 2012 (Table6). Unlike the amylose data set, starch granule size exhibited interactions among main effects. This limits opportunities to draw broad conclusions about the effects of variety, storage treatment, and environment. In a previous study, the CIS-susceptible cultivar Norchip had smaller starch granules when stored for 4 or 12 weeks at 4°C than when stored for the same amount of time at 12°C (Barichello et al. 1990). The CIS-resistant variety ND860-2 had more stable starch granules, with differences not detected until 24 weeks of cold storage. In our study, when tubers were stored at 6°C for 7 weeks, starch granules were no different in size than those from freshly harvested tubers. This held true even when CIS-resistant and CIS-susceptible varieties were analyzed separately. However, we did not compare starch granules of tubers stored at room temperature with those stored in a cooler. A large effect of environment on starch granule size has been reported (Johnston et al. 1968).

**Table 5:** Effect of potato variety on starch granule surface area.

Year	Variety	CIS	Area (*μ*m^2^)
2011	Atlantic	Susceptible	5263a
	M5	Resistant	4750ab
	Yukon Gold	Susceptible	3781bc
	ND860-2	Resistant	3317c
2012	Atlantic	Susceptible	5278a
	M5	Resistant	4850a
	ND860-2	Resistant	3841b
	Yukon Gold	Susceptible	3141c

Within a year, numbers followed by the same letter are not significantly different based on an LSD test, with*P* = 0.05. The CIS column indicates whether the variety is resistant or susceptible to cold-induced sweetening.

**Table 6:** Effect of field location on starch granule surface area in each of two production years.

Year	Location	Area (*μ*m^2^)
2011	Antigo	4790a
	Hancock	3766b
2012	Hancock	4904a
	Antigo	3651b

Within a year, numbers followed by the same letter are not significantly different based on an LSD test, with*P* = 0.05.

## Conclusions

Amylose content in tuber starch of potato varieties with resistance to cold-induced sweetening was higher than that of susceptible varieties. In contrast, starch granule size did not differ between resistant and susceptible varieties. Amylose content should be considered when developing and evaluating potato varieties for resistance to cold-induced sweetening.
